# Multiwavelength SERS of Magneto-Plasmonic Nanoparticles Obtained by Combined Laser Ablation and Solvothermal Methods

**DOI:** 10.1021/acsomega.3c08007

**Published:** 2023-12-14

**Authors:** Martynas Talaikis, Lina Mikoliunaite, Aikaterini-Maria Gkouzi, Vita Petrikaitė, Evaldas Stankevičius, Audrius Drabavičius, Algirdas Selskis, Remigijus Juškėnas, Gediminas Niaura

**Affiliations:** † Department of Organic Chemistry, Center for Physical Sciences and Technology (FTMC), Saulėtekio Av. 3, LT-10257 Vilnius, Lithuania; ‡ Department of Physical Chemistry, Faculty of Chemistry and Geosciences, Vilnius University, Naugarduko Str. 24, LT-03225 Vilnius, Lithuania; § Department of Laser Technologies, Center for Physical Sciences and Technology (FTMC), Savanorių Av. 231, LT-02300 Vilnius, Lithuania; ∥ Department of Characterization of Materials Structure, Center for Physical Sciences and Technology (FTMC), Saulėtekio Av. 3, LT-10257 Vilnius, Lithuania

## Abstract

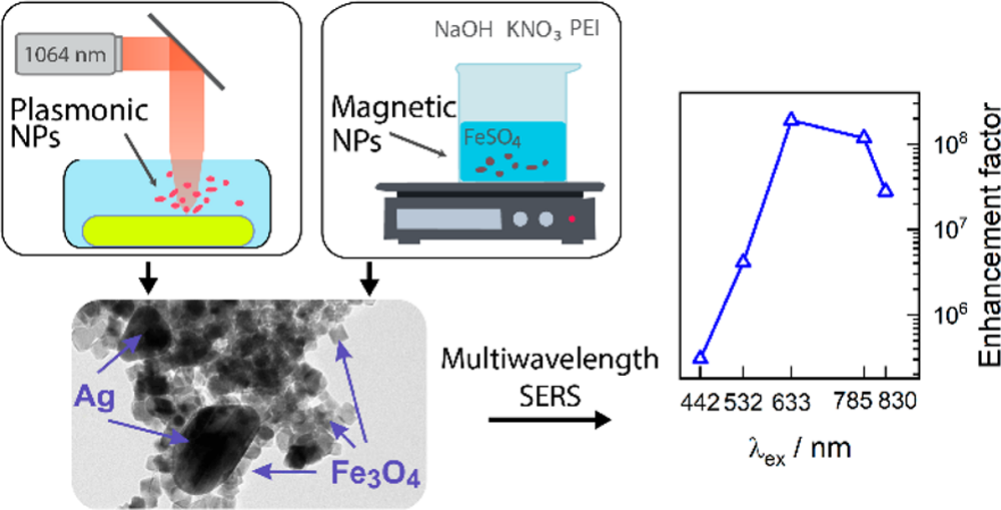

The present study introduces a novel method for the synthesis of magneto-plasmonic nanoparticles (MPNPs) with enhanced functionality for surface-enhanced Raman scattering (SERS) applications. By employing pulsed laser ablation in liquid (PLAL) to synthesize plasmonic nanoparticles and wet chemistry to synthesize magnetic nanoparticles, we successfully fabricated chemically pure hybrid Fe_3_O_4_@Au and Fe_3_O_4_@Ag nanoparticles. We demonstrated a straightforward approach of an electrostatic attachment of the plasmonic and magnetic parts using positively charged polyethylenimine. The MPNPs displayed high SERS sensitivity and reproducibility, and the magnetic part allowed for the controlled separation of the nanoparticles from the reaction mixture, their subsequent concentration, and their precise deposition onto a specified surface area. Additionally, we fabricated alloy based MPNPs from Ag_*x*_Au_100–*x*_ (*x* = 50 and 80 wt %) targets with distinct localized surface plasmon resonance (LSPR) wavelengths. The compositions, morphologies, and optical properties of the nanoparticles were characterized by using transmission electron microscopy (TEM), scanning electron microscopy (SEM), X-ray diffraction (XRD), UV–vis spectroscopy, and multiwavelength Raman spectroscopy. A standard SERS marker, 4-mercaptobenzoic acid (4-MBA), validated the enhancement properties of the MPNPs and found an enhancement factor of 2 × 10^8^ for the Fe_3_O_4_@Ag nanoparticles at 633 nm excitation. Lastly, we applied MPNP-enhanced Raman spectroscopy for the analysis of the biologically relevant molecule adenine and found a limit of detection of 10^–7^ M at 785 nm excitation. The integration of PLAL and wet chemical methods enabled the relatively fast and cost-effective production of MPNPs characterized by high SERS sensitivity and signal reproducibility that are required in various fields, including biomedicine, food safety, materials science, security, and defense.

## 1 Introduction

Magneto-plasmonic nanoparticles (MPNPs) represent a novel category of hybrid nanomaterials that combine the intrinsic properties of magnetic (such as Fe_3_O_4_ and Co) and plasmonic (such as Au and Ag) components to enhance their functionalities.^1−5^ These nanoparticles (NPs) are exploited in various fields of biomedicine, sensing, catalysis, light harvesting, magnetic resonance imaging, and surface-enhanced Raman scattering (SERS) applications.^6,7^ SERS is a label-free technique that delivers high specificity and sensitivity for molecules adjacent to plasmonic surfaces. It provides in-depth information on the chemical bonds and enables a comprehensive understanding of the molecular structure. SERS is based on the localized surface plasmon resonance (LSPR) effect, which is the collective oscillation of free-electron plasma excited by an incident electromagnetic radiation. The synergetic integration of magnetic and plasmonic properties effectively resolves some critical challenges of SERS by increasing the homogeneous nanoparticle distribution under an external magnetic field, resulting in a diminished “coffee-ring” effect and increased SERS signal reproducibility.^8−10^ Magnetism also allows for the spatial manipulation of nanoparticles, facilitating their targeted placement and concentration on a specific surface area.^11^

There are various methods, broadly categorized as bottom-up (synthesis from atoms) and top-down (synthesis from the bulk material), for the generation of nanoparticles. The former encompasses chemical reduction, sol–gel, hydrothermal, and biological synthesis methods, whereas the latter includes methods such as lithography, etching, laser thermal modification, and pulsed laser ablation in liquid (PLAL).^12−16^ PLAL is a rapid, versatile, and simple method that uses no additional chemicals and results in in situ, ultrapure nanoparticle dispersion.^17^ In PLAL, the target material is submerged in a liquid medium and subjected to high-intensity pulsed laser irradiation, which leads to the rapid melting and vaporization of the material, followed by the formation of a cavitation bubble. The collapse of the cavitation bubble results in the ejection of molten or vaporized material into the surrounding liquid, where it condenses into nanoparticles.^18,19^ PLAL offers partial control over the nanoparticle size, size distribution, shape, and aggregation through the modulation of the parameters, including the laser wavelength, pulse width and rate, and fluence.^12^ Additional factors such as the liquid medium, target, and external fields also strongly affect the final result.^13,20,21^

The ablation medium plays a crucial role in regulating the product outcome. A chemical analysis of Au nanoparticles produced by PLAL in saline solution revealed the presence of halide ions on the nanoparticle surfaces, supporting a hypothesis that halogen anions contribute to colloidal stability by carrying a negative charge.^22^ In contrast, other studies of the wet chemical preparation of plasmonic nanoparticles found inorganic salt to induce aggregation and hot-spot formation that strongly contribute to SERS sensitivity.^23−25^ Moreover, even certain cation–anion pairs, specifically LiCl, have been recognized as particularly effective in increasing the SERS signal.^26^ Hot-spot formation alone cannot be attributed to the increased signal, as the chlorides have also promoted the SERS signal for the roughened Ag surface.^26^ Halides form surface complexes characterized by low-lying excited states that readily undergo photoinduced charge transfer from the target molecule and contribute to SERS enhancement via a chemical enhancement mechanism.^27^ They also disturb the electric double layer and change the electrostatic interactions between the target molecule and the surface.

There are several approaches for generating hybrid bimetallic nanoparticles by PLAL, e.g., the sequential ablation of two metal targets in the same liquid medium, which leads to the mixing and coalescence of two different material nanoparticles, although the quality of the process may sometimes be limited.^28^ Another way is to use layered thin films of different metals, which results in alloyed and complex nanostructures as the core–shell.^3,29−32^ However, the alloying and dual structure formation are challenging to manage, and PLAL more often results in a mixture of different nanoparticle types, which translates into poor SERS enhancement. For example, MPNPs prepared by Fe and Au multilayer laser ablation exhibited an enhancement factor of 5.8 × 10^4^ for the 4-mercaptobenzoic acid (4-MBA) reporter molecule.^31^ Moreover, in a bimetallic alloy having Au/Ag and Fe components, the LSPR was found to sharply decline with increasingly higher Fe concentrations (above approximately 15%). Therefore, only a narrow concentration window of plasmonic material is practical for SERS.^33,34^ Another issue with MPNP production using PLAL is that the ablation of an iron target is limited to organic solutions because ablation in water results in the formation of weak magnetization iron oxides, such as maghemite.^32^ Organic solutions, however, contaminate the plasmonic nanoparticles with carbon species originating from the laser-induced thermal decomposition of the solvent.^31^

In this study, we propose an MPNP synthesis method in which the plasmonic part is obtained by PLAL in saline water and the magnetic part is obtained by a wet chemistry synthesis method. Polyethylenimine mediates the nanoparticle attachment and formation of the hybrid MPNP structure. Such an approach allows one to obviate the use of additional reducing and stabilizing agents that are typically necessary for plasmonic NPs and achieve a chemically pure colloid material. The magnetic nanoparticle synthesis, in contrast, relies on inorganic reagents and polyethylenimine (PEI), whose function is to attach the magnetic and plasmonic parts, resulting in hybrid Fe_3_O_4_@Au or Fe_3_O_4_@Ag nanoparticles. The enhanced SERS sensitivity and signal reproducibility of MPNPs compared to those of conventional plasmonic nanoparticles have already been proven.^9,10,35^ In this study, the MPNPs were probed using multiwavelength SERS and the spectroscopic marker molecule 4-MBA. We also show the possibility of forming MPNPs from Ag_*x*_Au_100–*x*_ alloy targets (*x* = 50 and 80 wt %), which adds another dimension of fine-tuning the LSPR frequency according to one’s needs. Finally, we apply MPNP-enhanced Raman spectroscopy to analyze the biologically relevant adenine molecule.

## 2 Materials and Methods

### 2.1 Synthesis of Plasmonic and Magneto-Plasmonic Nanoparticles

Plasmonic nanoparticles were obtained by laser ablation in liquid of Ag, Au (Micro to Nano, Haarlem, Netherlands), Ag_80_Au_20_, and Ag_50_Au_50_ (Testbourne Ltd., U.K.) targets (subscripts indicate wt %). A nanosecond laser (Ekspla, Lithuania) operating at a wavelength of 1064 nm with an average power of 7 W, a pulse duration of 10 ns, and and a 10 kHz repetition rate was used in the generation process. The diameter of the Gaussian beam at the surface was ∼100 μm (at the level of 1/e^2^). The laser fluence was ∼17.8 J/cm^2^. The hatch (distance between the laser scan lines) was chosen to be 50 μm, and the scanning speed of the beam was 500 mm/s. Laser ablation was performed in various concentrations of aqueous KCl solution using a 40 mL capacity of Petri dishes. The ablated nanoparticles were immediately used for modification with magnetic nanoparticles.

The magnetic nanoparticle synthesis is described elsewhere.^9^ Briefly, 0.175 g of FeSO_4_·7H_2_O (Carl Roth GmbH, Karlsruhe, Germany) was dissolved in 20 mL of Ar-purged water. Then, 2.5 mL of 2 M KNO_3_ (Reachem, Bratislava, Slovakia), 2.5 mL of 1 M NaOH (Carl Roth GmbH, Karlsruhe, Germany), and 5 mL of 8 mg/mL PEI (*M*_w_ = 2500; Sigma-Aldrich, Steinheim, Germany) were added and magnetically stirred under a N_2_ gas flow at 90 °C for 2 h. After the solution cooled, the magnetic nanoparticles were triple-washed with deionized water and decorated with either silver, gold, or silver–gold alloy nanoparticles according to the procedure described elsewhere.^9^

**Scheme 1:**
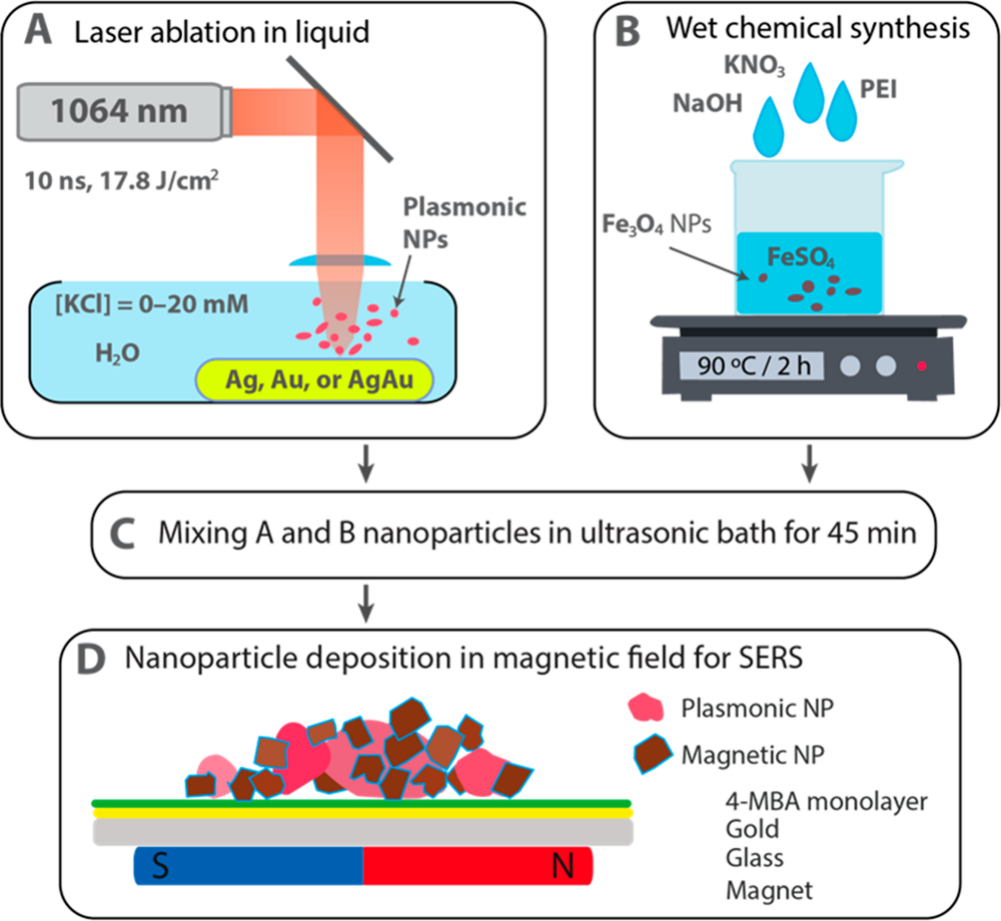
Flowchart of Magneto-Plasmonic Nanoparticle Preparation and Application for SERS (A) Laser ablation of plasmonic metal target in water containing 0–20 mM KCl. (B) Chemical synthesis of magnetic nanoparticles. (C) Mixing of A and B colloidal solutions and subjecting them to an ultrasound treatment for 45 min to form MPNP aggregates. (D) MPNP deposition under an external magnetic field on a gold film-adsorbed 4-MBA self-assembled monolayer, followed by SERS characterization.

To obtain hybrid nanoparticles (MPNPs), 10 mL of the plasmonic nanoparticles was mixed with 0.8 mL of the Fe_3_O_4_ colloidal solution. After a 45 min ultrasound treatment, the decorated nanoparticles (termed Fe_3_O_4_@Ag, Fe_3_O_4_@Au, Fe_3_O_4_@Ag_80_Au_20_, and Fe_3_O_4_@Ag_50_Au_50_) were collected by using a strong magnet and washed with distilled water three times. The final colloid solution was resuspended to 5 mL. The magneto-plasmonic nanoparticle preparation method is illustrated in Scheme 1. All preparations were conducted using deionized water (18.2 MΩ cm) from the Direct-Q 3 UV purification system (St. Louis, Missouri, U.S.).

### 2.2 Nanoparticle Characterization

Thin Au films (∼150 nm) were magnetron-sputtered on microscope glass slides by using a PVD 75 system (Lesker, U.K.). Afterward, the slides were incubated in 4-mercaptobenzoic acid (4-MBA) (99%; Sigma-Aldrich, St. Louis, Missouri, U.S.) in an ethanol solution (0.2 mM) overnight. The MPNPs were deposited in a magnetic field on a 4-MBA self-assembled monolayer and allowed to dry. The magnetic field was removed prior to the SERS measurements. SERS was carried out using a multiwavelength inVia Raman spectrometer (Renishaw, Wotton-under-Edge, U.K.) equipped with a confocal Leica microscope and a thermoelectrically cooled (−70 °C) CCD (charge-coupled device) camera. Laser excitation sources were used in combination with diffraction gratings as follows: 442 nm (2400 lines/mm), 532 and 633 nm (1800 lines/mm), 785 nm (1200 lines/mm), and 830 nm (830 lines/mm). The laser radiation was focused on the sample using a 50×/0.75 NA (numerical aperture) objective lens (Leica). SERS spectra were obtained by averaging 100 spectra from different sample locations with 25 s of integration each. SERS enhancement factors (EFs) for different laser excitations of the dominant 4-MBA spectral modes near 1073 and 1581 cm^–1^ were calculated according to a procedure detailed elsewhere^36,37^ and by taking the geometric parameters of 4-MBA from the literature.^38^ In short, the EF calculation procedure was as follows. EF = (*I*_SERS_*N*_R_)/(*I*_R_*N*_SERS_), where *I*_SERS_ and *I*_R_ are the intensities of the SERS and Raman spectral bands at 1585/1595 cm^–1^, respectively, and *N*_R_ and *N*_SERS_ are the number of molecules excited in the Raman and SERS measurements, respectively.

The SERS spectra of adenine (≥99%; Sigma-Aldrich, St. Louis, Missouri, U.S.) were collected using a HyperFlux PRO Plus Raman spectrometer (Tornado Spectral Systems, Mississauga, Ontario, Canada) equipped with a thermoelectrically cooled CCD camera, a fiber-optic cable, and a 785 nm wavelength laser source that produced a 105 μm diameter spot on the sample. First, 3 μL of the as-prepared nanoparticle solution was dried on a steel substrate under an external magnetic field. Then, 3 μL of the desired concentration of an aqueous adenine solution was cast onto the top and dried. After that, the magnet was removed, and the samples were probed using a laser power of 70 mW at the sample (0.7 kW/cm^2^) and an accumulation time of 10–300 s. Several consecutive measurements were carried out and scrutinized on possible spectral changes to ensure there was no photothermal damage to the sample.

The optical properties of the plasmonic and hybrid nanoparticles were measured by using an ultraviolet–visible (UV–vis) spectrometer (Cary 5000, Agilent, U.S.). Size and composition characterizations were performed with high-resolution transmission electron microscopy (HRTEM; Tecnai G2 F20 X-TWIN, FEI, Hillsboro, Oregon, U.S.), scanning electron microscopy (SEM; Helios Nanolab 650, FEI, Netherlands), and X-ray diffraction (XRD; SmartLab, Rigaku, Tokyo, Japan).

## 3 Results and Discussion

### 3.1 Characterization of Plasmonic Nanoparticles

**Figure 1:**
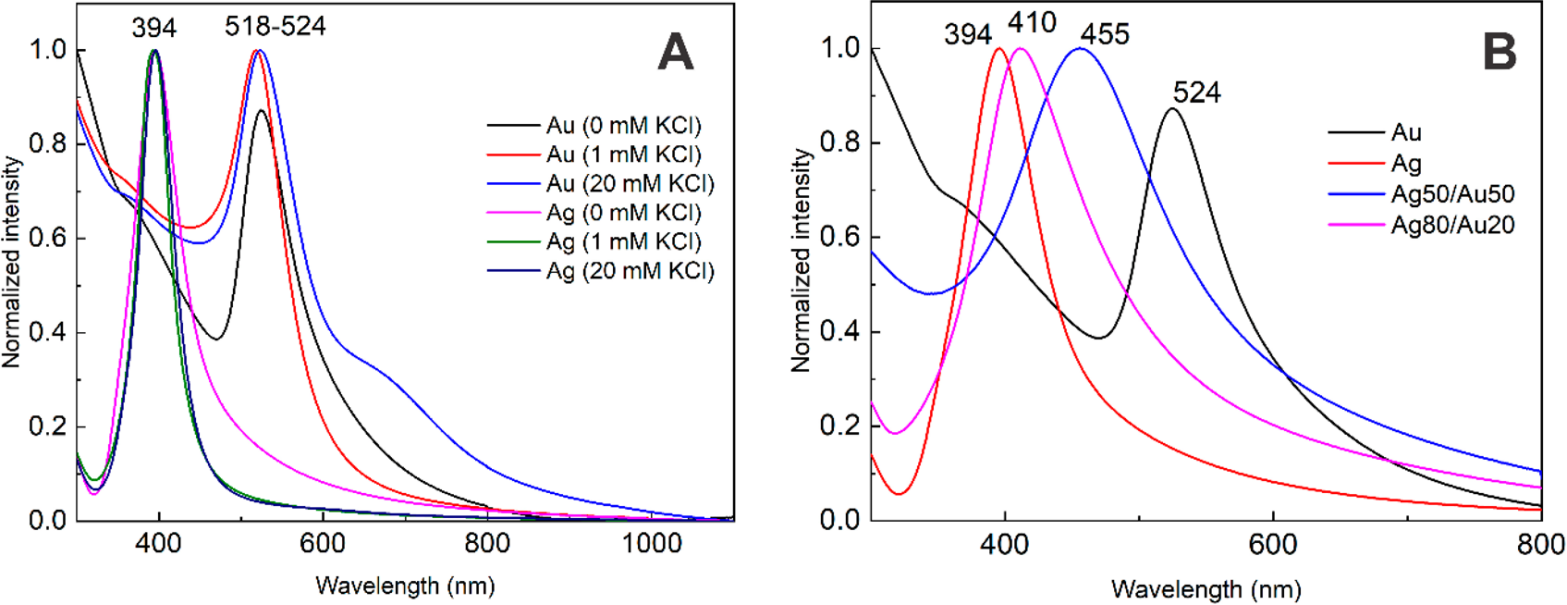
UV–vis spectra of nanoparticles obtained by PLAL of (A) Au and Ag targets in 0–20 mM KCl solutions and (B) Ag, Au, Ag_80_Au_20_, and Ag_50_Au_50_ targets in pure water. The spectra are normalized to 1 for a better comparison.

**Figure 2:**
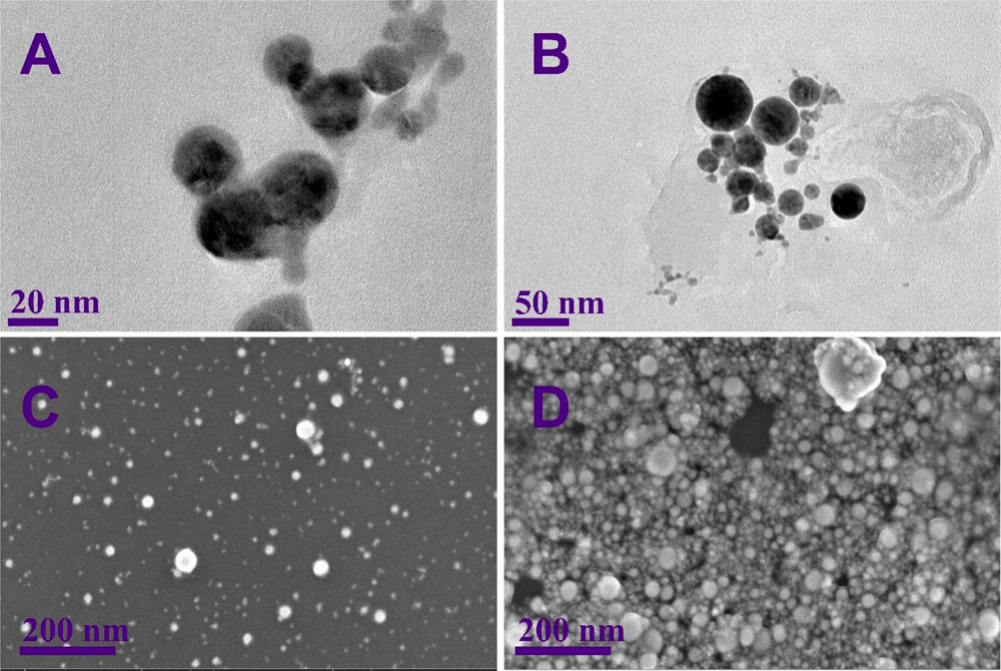
(A, B) TEM and (C, D) SEM images of plasmonic nanoparticles obtained from (A, C) Ag_80_Au_20_ and (B, D) Ag_50_Au_50_ targets. KCl concentration was 1 mM.

The plasmonic nanoparticles were prepared by laser ablation of Au, Ag, and two Ag/Au alloy targets in saline water. The rationale for using PLAL in saline water is as follows: halide ions (i) increase colloidal stability, preventing aggregation, (ii) contribute to increased SERS sensitivity, and (iii) help to displace weakly adsorbed species and prevent nanoparticle (NP) surface fouling, leading to a more reproducible SERS signal. Figures 1 and 2 show the UV–vis and electron microscopy characterizations of the plasmonic NPs, respectively. The LSPR peak for the Ag NPs was observed at 394 nm and was independent of the Cl^–^ concentration in the ablation medium. While a narrow size distribution, evidenced by the width of the LSPR peak, was visible for each saline water-ablated NP batch, the ones ablated in pure water exhibited an asymmetric tail in the higher wavelength region, indicating a population of NPs with increased size or aggregation.

The Au NPs were more sensitive to the Cl^–^ concentration than the Ag NPs. The detected peak blueshift was about 6 nm for the Au NPs when the chloride concentration in the solution was altered from 1 to 20 mM. We also observed a broadening of the absorption peak and an emergence of a second peak near 670 nm, related to the NP aggregates, in the case of 20 mM chloride. Increasing the concentration to 30 mM led to rapid NP aggregation and sedimentation (Figure S1), rendering a weak absorption spectrum. Hence, these NPs were excluded from further investigation.

The UV–vis spectra of the NPs obtained from Ag and Au alloy targets in pure water are presented in Figure 1B. Both compositions show only one LSPR peak, indicating there was no segregation into monometallic or core–shell NPs. The detected absorption maximum shift is consistent with the target composition. Interestingly, the alloy target NPs exhibited greater absorption peak full width at half-maximum (fwhm) values: specifically, the fwhm values were 73 and 136 nm for the Ag_80_Au_20_ and Ag_50_Au_50_ NPs, respectively, while they were 59 and 62 nm for the Au and Ag NPs, respectively. Such a broadening for bimetallic alloy NPs was observed previously.^39,40^

The nanoparticles were also characterized by using SEM and TEM techniques (Figure 2). As can be seen from the SEM images, the nanoparticles vary in size from several nanometers to 50–70 nm. However, the spherical form is maintained in most of the nanoparticles. From the TEM images, the size of the nanoparticles can be determined more precisely. Sizes range from 10 to approximately 100 nm, and it can be seen that the nanoparticles tend to aggregate. In addition to this, the nanoparticles seem to be homogeneous without separate core–shell or Janus-type compositions. This was also confirmed by energy dispersive X-ray (EDX) analysis. EDX analysis indicated that the Ag/Au alloy target composition was retained in the NPs, and no anisotropic separation of the two metals was detected (Figure S2). XRD measurements also confirmed the composition of the NPs. Although the crystal lattice parameters for these samples are similar, 4.0790 and 4.0786 Å, respectively, the peaks of the Ag_80_Au_20_ and Ag_50_Au_50_ NPs are slightly shifted (Figure S3).

### 3.2 Characterization of Hybrid Magneto-Plasmonic Nanoparticles Consisting of Magnetite and Gold or Silver

**Figure 3:**
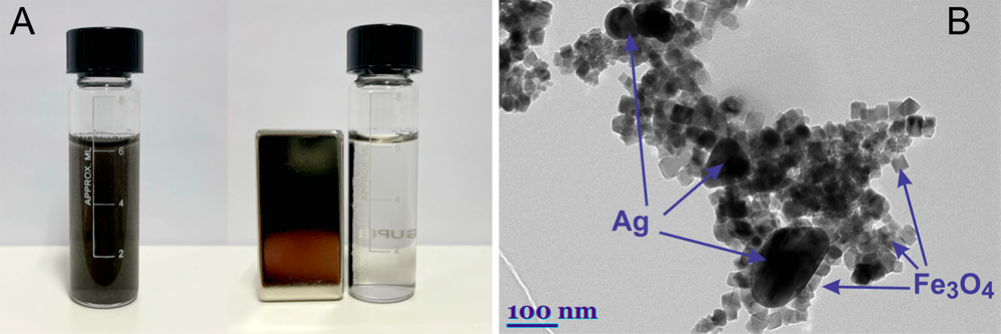
(A) Photographs of the MPNP solution (Fe_3_O_4_@Ag) before and after concentration using a neodymium magnet. (B) TEM image of MPNPs. Magnetite (Fe_3_O_4_) nanoparticles are cubic in shape and light gray; Ag nanoparticles are round and dark gray.

The magnetic nanoparticles were synthesized using polyethylenimine (PEI), resulting in magnetite NPs with a positive surface charge, whereas plasmonic NPs are typically negatively charged. Thus, the formation of hybrid magneto-plasmonic NPs is governed by the electrostatic pull, and it was accomplished by simply mixing the magnetic and plasmonic NPs solutions. To ensure complete association, we used an ultrasonic bath for 45 min. Subsequently, the hybrid NPs were isolated by using a magnet and were washed three times with distilled water to remove any impurities. Figure 3A presents a bottle with the nanoparticles dispersed in water. When a magnetic field was applied, all of the nanoparticles were attracted to the side of the bottle, and only a transparent solute remained. Such an observation is evidence that the plasmonic nanoparticles were strongly attached to the magnetic nanoparticles, and they move together in the solution. This was also confirmed by the TEM image of the hybrid NPs (Figure 3B). The magnetic nanoparticles were uniform cubic structures that were 50 nm in size, while the plasmonic nanoparticles were spheres of various sizes. In the TEM image, dark structures represent the silver nanoparticles, while light gray cubic forms and surrounding plasmonic parts are the magnetic nanoparticles. In TEM images, the darkness of the color depends on the weight of the atom. Because the Ag atom is heavier than the Fe atom, a color difference appears.

**Figure 4:**
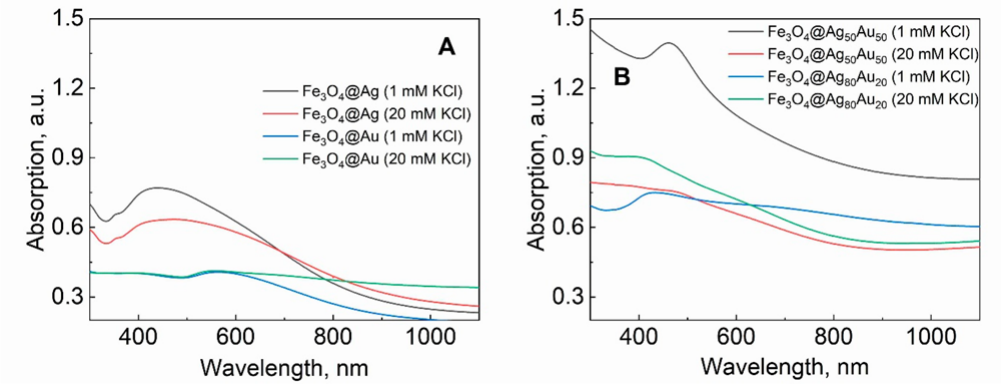
UV–vis absorption spectra of hybrid magneto-plasmonic nanoparticles. The plasmonic part was obtained from (A) pure Au and Ag and (B) alloyed Ag_80_Au_20_ and Ag_50_Au_50_ targets by PLAL in saline water.

The UV–vis spectra of the hybrid NPs (Figure 4) show less pronounced absorptions compared to the plasmonic NP spectra (Figure 1), mostly due to the increased light scattering by magnetite at shorter wavelengths. In general, due to interactions with magnetite, the plasmonic absorption peak tended to shift to longer wavelengths and broaden. For example, for the Ag and Au MPNPs, the maxima were found in the 400–500 nm range and near 550 nm, respectively, compared to 394 and ca. 520 nm for the Ag and Au NPs, respectively. This shifting can be ascribed partially to the high dielectric constant of magnetite affecting the plasmonic properties of the Ag and Au NPs.^3^ Unfortunately, the LSPR absorption peaks for the hybrid alloy NPs were very weakly pronounced.

The wet chemical synthesis approach, outlined in our earlier work, generates magnetite cubes averaging around 50 nm in diameter.^9^ The TEM image in Figure 3B demonstrates a uniform magnetite distribution. Conversely, pulsed laser ablation in liquid results in plasmonic nanoparticles of a higher average size with a broader distribution. Hence, the resultant MPNPs comprise a magnetite nanoparticle network with multiple larger plasmonic nanoparticles trapped inside due to electrostatic interactions. This arrangement facilitates the formation of hot-spot junctions, contributing to a significant enhancement in the Raman signal intensity; this may also be the reason for the redshifted LSPR peaks in the absorption spectra. No evidence for possible core–shell structure formation was found.

### 3.3 SERS of Magneto-Plasmonic Nanoparticles Consisting of Magnetite and Gold or Silver

**Figure 5:**
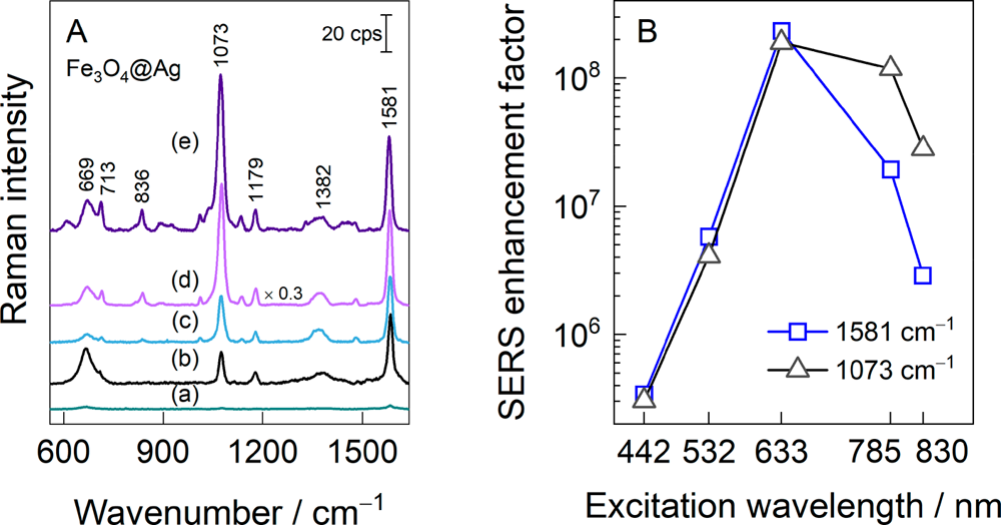
(A) The SERS spectra of the 4-MBA molecule were amplified by utilizing hybrid Fe_3_O_4_@Ag nanoparticles at various laser excitations and power levels: (a) 442 nm (53 μW), (b) 532 nm (450 μW), (c) 633 nm (4.7 μW), (d) 785 nm (90 μW), and (e) 830 nm (160 μW). The plasmonic part of the hybrid nanoparticles was obtained by laser ablation of the Ag target in a 20 mM KCl aqueous solution. (B) The excitation-wavelength-dependent SERS enhancement factor calculated for the 1073 and 1581 cm^–1^ modes of 4-MBA.

The SERS sensitivity of the hybrid MPNPs deposited under an external magnetic field was probed using a 4-MBA self-assembled monolayer adsorbed on an atomically flat Au surface. This molecule does not possess a resonant Raman enhancement effect in the visible spectral region and strongly adsorbs onto metal surfaces, providing opportunity for the reliable probe of SERS substrates.^41^Figure 5A shows how the SERS spectra were amplified by the Fe_3_O_4_@Ag NPs obtained by PLAL in a 20 mM KCl aqueous solution and excited at different wavelengths. Most spectral modes were assigned to 4-MBA molecules, whereas the broad spectral feature near 669 cm^–1^ is evidence of the presence of magnetite.^42^ No vibrational bands from maghemite (γ-Fe_2_O_3_) or hematite (α-Fe_2_O_3_) were observed in the SERS spectra, indicating that the magnetic part of the hybrid nanoparticles was composed from a pure magnetite structure. Figure 5B presents the SERS enhancement factors (EFs) calculated at different laser excitation wavelengths for the two most prominent spectral modes of 4-MBA at 1073 and 1581 cm^–1^. These modes are assigned to ν_12_ ring breathing vibration coupled with C–S stretching and ν_8a_ ring vibration, respectively.^5,43,44^ Fe_3_O_4_@Ag exhibits optimal performance within the red spectral region. The highest SERS EFs were found for 633 nm excitation, having the values of 1.9 × 10^8^ and 2.3 × 10^8^ for the 1073 and 1581 cm^–1^ modes, respectively. The excitation in the blue region (442 nm) still showed a moderate enhancement of approximately 3 × 10^5^.

**Figure 6:**
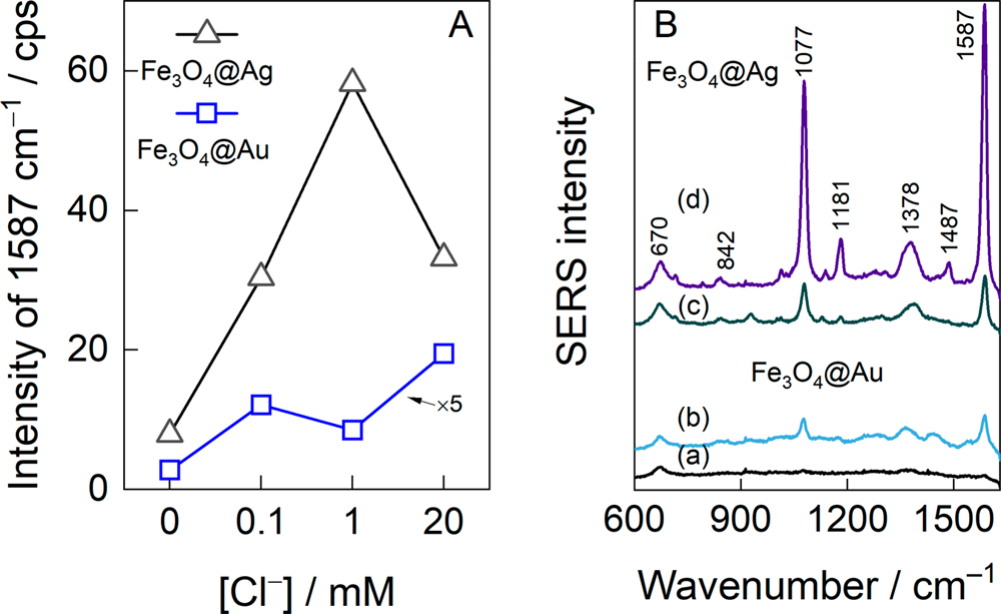
(A) Relative SERS intensity of the 1587 cm^–1^ spectral mode of the 4-MBA monolayer enhanced by Fe_3_O_4_@Ag and Fe_3_O_4_@Au NPs. The plasmonic nanoparticles were obtained from solutions containing varied KCl concentrations. Notice the scaling of the Fe_3_O_4_@Au dependency. (B) 4-MBA monolayer spectra excited with MPNPs obtained from (a, c) 0 and (b, d) 20 mM KCl solutions using PLAL. The results are averages of 100 measurements from different spots on a sample. Laser excitation wavelength: 633 nm, power: 45 μW, and integration time: 25 s.

Figure 6A addresses the influence of potassium chloride on the PLAL outcome and the SERS sensitivity. Compared to the pure-water-ablated Fe_3_O_4_@Ag NPs, the addition of 0.1, 1, and 20 mM KCl in the ablation medium increased the SERS sensitivity at 633 nm excitation by 4.3, 11.7, and 7.4 times, respectively. The Fe_3_O_4_@Au NPs, in contrast, showed a much poorer performance in general. Nonetheless, chloride-related signal intensification was also evident, and the calculated factors were 4.4, 3.1, and 7 times higher than for the nanoparticles prepared without chloride. The SERS sensitivity increase induced by halide adsorption on the Au/Ag NPs surface was hypothesized to be related to the SERS chemical mechanism and aggregation of plasmonic NPs (electromagnetic mechanism).^27^

Notably, the prepared MPNPs were stable during prolonged storage at room temperature and were functional after at least seven months after production. No segregation occurred; only rapid sedimentation was observed, which could be readily dispersed by shaking or an ultrasonic bath.

Using saline-water-prepared MPNPs also affected the relative intensities of the 4-MBA spectral pattern (Figure 6B). For example, the modes at 1181 and 1487 cm^–1^ strengthened, and the mode at 1378 cm^–1^ decreased in intensity compared to the spectra recorded for the nanoparticles prepared in the presence of 20 mM KCl with pure-water-prepared NPs. The 1181 cm^–1^ mode is related to ring in-plane C–H deformations β(CH),^45^ whereas the mode at 1487 cm^–1^ is assigned to ν(C–C) + γ(CH), and the mode at 1378 cm^–1^ is related to the surface-bound carboxylate group stretching vibration ν_s_(CCO^–^).^44,46^ The observed differences in relative intensities are related to structural changes within the self-assembled monolayer. These changes might be attributed to the co-adsorption of chloride ions, potentially originating from the nanoparticles, onto the gold substrate alongside the 4-MBA monolayer.

### 3.4 Characterization of Hybrid Magneto-Plasmonic Nanoparticles Consisting of Gold and Silver Alloys

**Figure 7:**
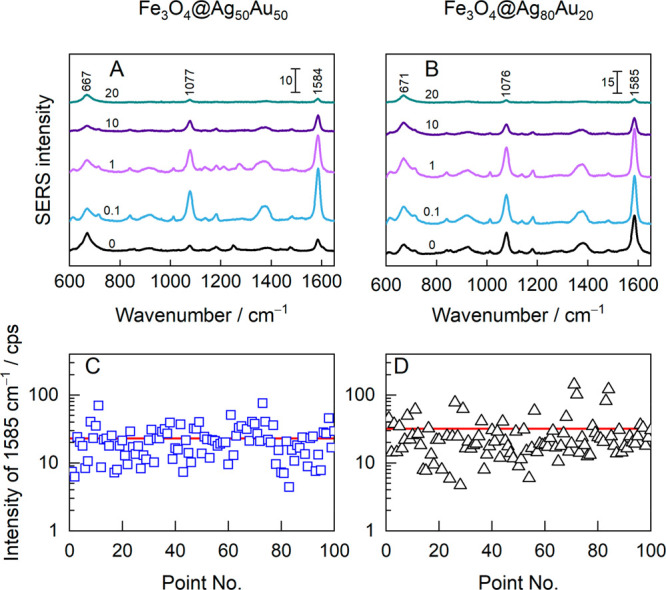
Spectroscopic characterization of Ag_*x*_Au_100–*x*_ alloy MPNPs. Average SERS spectra of 4-MBA monolayer excited using (A) Fe_3_O_4_@Ag_50_Au_50_ and (B) Fe_3_O_4_@Ag_80_Au_20_ NPs that were prepared by PLAL in 0–20 mM KCl aqueous solutions (salt concentrations are indicated above the spectra). (C, D) The corresponding SERS intensity distributions of the 4-MBA 1585 cm^–1^ mode at 100 individual spots on the surface. The red line indicates the average value. The MPNPs were prepared in a 0.1 mM KCl solution. Laser excitation was 633 nm (45 μW), and the integration time was 25 s.

**Figure 8:**
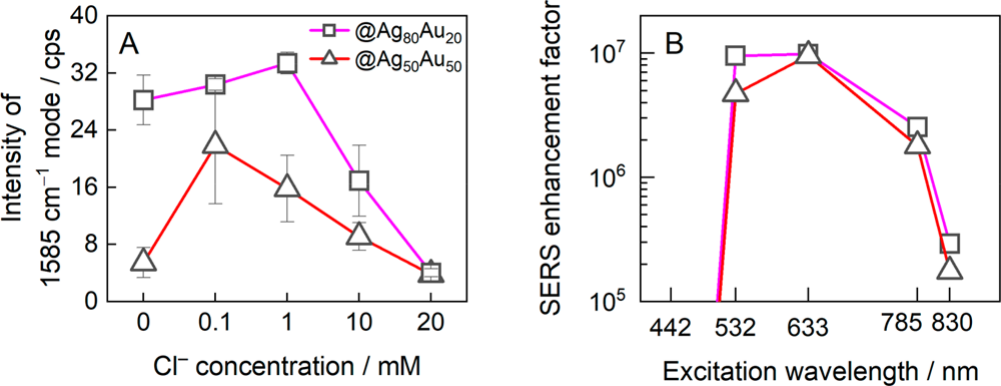
(A) The dependence of the SERS intensity of the 1585 cm^–1^ mode on the Cl^–^ concentration in the ablation medium. Laser excitation was 633 nm (45 μW), and the integration time was 25 s. (B) SERS enhancement factor for Fe_3_O_4_@Ag_80_Au_20_ and Fe_3_O_4_@Ag_50_Au_50_ NPs calculated at different excitation wavelengths and based on the 1585 cm^–1^ intensity (KCl concentration was 0.1 mM).

One of the advantages of using bimetallic Ag/Au alloy NPs is the ability to fine-tune the plasmonic properties by changing the metal ratio, as the two metals are entirely miscible (Figure 1B). The LSPR maximum can be easily varied from ca. 400 to 530 nm for spherical plasmonic NPs,^14^ while in conjugation with magnetite, it shifts to longer wavelengths. Panels A and B of Figure 7 show the SERS spectra of 4-MBA excited by using hybrid alloy NPs, specifically Fe_3_O_4_@Ag_50_Au_50_ and Fe_3_O_4_@Ag_80_Au_20_, respectively. The corresponding intensity distributions of the 1585 cm^–1^ mode acquired from 100 spots on the surface are shown in panels C and D of Figure 7. In Figure 8A, the intensity of the dominant 1585 cm^–1^ mode is plotted against the KCl concentration in the ablation medium. An increase in KCl concentration from 0 to 0.1 mM led to a 4-fold spectral intensity increase in the case of the Fe_3_O_4_@Ag_50_Au_50_ NPs, followed by a reduction of the SERS signal at higher concentrations. In contrast, the SERS sensitivity remained relatively stable within the 0–1 mM KCl range for the Fe_3_O_4_@Ag_80_Au_20_ nanoparticles, yet it was markedly higher compared to the Ag_50_Au_50_ alloy. The optimal KCl concentration in the ablation medium lies between 0.1 and 1 mM for both alloy compositions; concentrations exceeding 10 mM had a detrimental effect on SERS sensitivity. We further employed the alloy MPNPs ablated in 0.1 mM KCl solution to achieve multiwavelength SERS enhancement factors (Figure 8B). Both compositions displayed almost the exact same dependency on the excitation wavelength, with EF maxima of 9.6 × 10^6^ and 9.9 × 10^6^ for the 50/50 and 80/20 compositions of the Ag/Au alloy, respectively, at 633 nm. The EFs were about 1 order of magnitude lower than the EF found for Fe_3_O_4_@Ag, and they noticeably shifted toward shorter wavelengths. In the case of the alloy MPNPs, shifting from 633 to 532 nm excitation did not show a considerable decrease in the SERS EF compared to Fe_3_O_4_@Ag, which showed a clear EF reduction by approximately 45×. Finally, only very weak SERS spectra were obtained at 422 nm excitation.

### 3.5 SERS Analysis of Adenine Adsorbed at MPNPs

**Figure 9:**
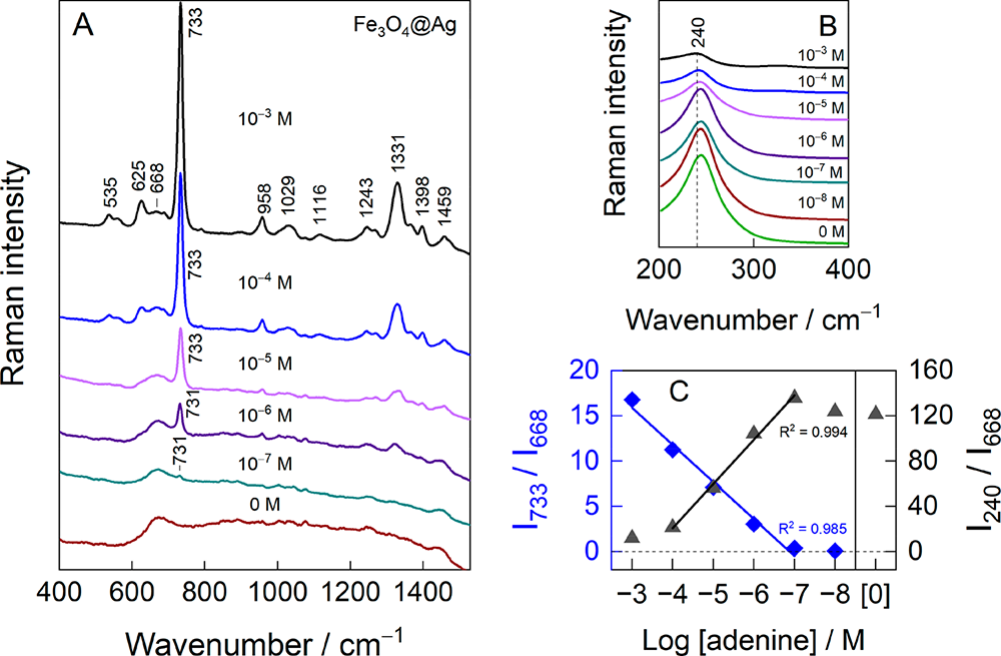
SERS spectra of varying concentrations of adenine enhanced using Fe_3_O_4_@Ag nanoparticles prepared in a Cl^–^-containing solution in (A) the 490–1530 cm^–1^ range and (B) the Ag–Cl stretching region. Laser excitation was 785 nm (0.2 kW/cm^2^). (C) Concentration-dependent relative intensities of the adenine ring breathing mode at 733 cm^–1^ and ν(Ag–Cl) at 240 cm^–1^ normalized to the ν(Fe–O) mode of magnetite. “[0]” denotes pristine nanoparticles without adsorbate.

In evaluating the SERS efficacy of the Fe_3_O_4_@Ag nanoparticles synthesized in a chloride-containing solution, adenine of varying concentrations was used as a biologically relevant probe molecule.^41^Figure 9A shows the vibrational modes associated with the molecular structure of adenine. The most intense band at 731–733 cm^–1^ is associated with ring breathing motion, and its position is characteristic of Ag surface-adsorbed molecules rather than bulk adenine, whose Raman band appears around 723 cm^–1^.^31,47^ No such band was observed in the given concentration window of our experiment. Other notable bands included 625 cm^–1^, assigned to ν(C–C) mixed with the in-plane bending of adenine rings; 958 cm^–1^, related to NH_2_ rocking coupled with C–N stretching; 1331 cm^–1^, assigned to ν(C–N) and β(CH); and 1398 cm^–1^, related to ν(C–C) and β(CH).^31,47−49^ The mode at 668 cm^–1^ is associated with the stretching motion of Fe–O of magnetite. No additional iron oxide phases, such as maghemite (broad spectral features at 350, 500, and 700 cm^–1^) or hematite (290 and 412 cm^–1^), were recognized from the spectra.^50^ The low-frequency region (Figure 9B) contained a silver–chloride stretching mode at 240 cm^–1^.

The intensity of the 733 cm^–1^ mode decreased with the adenine concentration, and the determined detection limit was 10^–7^ M. Table 1 presents a literature survey of the SERS detection limits for adenine utilizing a range of nanoparticles. Depending on the nanoparticles, the SERS detection limit of adenine varies within the range from 10^–6^ to 10^–14^ M.^51−66^ In this study, the detection limit appears at the higher adenine concentration range compared with the values reported in the literature. One of the factors influencing the detection limit is the incorporation of the non-SERS-active magnetite component in the MPNPs. An increasing adenine concentration resulted in a diminishing ν(Ag–Cl) intensity, which hints at the competitive replacement of chlorides from the nanoparticle surface. Interestingly, we observed a noticeable shift from 731 to 733 cm^–1^ when the adenine concentration changed from 10^–6^ to 10^–5^ M, accompanied by a −4 cm^–1^ shift in the Ag–Cl stretching mode frequency in a much broader concentration range; such a concentration dependency points to interactions between co-adsorbed adenine and chloride on the surface. The intensity dependencies of these two modes, when normalized to magnetite’s ν(Fe–O) band and plotted against the logarithm of the adenine concentration, exhibit a linear trend in the range from 10^–3^ and 10^–4^ to 10^–7^ M (Figure 9C).

**Table 1:** Literature Survey of SERS Detection Limits of Adenine^a^

SERS substrate	detection limit (M)	excitation wavelength (nm)	reference
Fe_3_O_4_@Ag	10^–7^	785	present work
magnetic microspheres decorated with Ag nanoparticles	>10^–7^	633	(51)
Ag nanoparticle clusters	10^–11^	532	(52)
GO coated with Ag NPs	10^–14^	785	(53)
Au nanorods and Ti_3_C_2_T_*x*_ composites	10^–9^	633	(54)
Ag nanocubes coated with SiO_2_ and deposited on paper	8.9 × 10^–10^	532, 633	(55)
Au NP and carbon nanosheet hybrids	3 × 10^–7^	785	(56)
halloysite nanotubes functionalized with Au and Ag NPs	1.6 × 10^–8^	785	(57)
Ag nanorod arrays	10^–7^	785	(58)
Au film-coated glass capillary	10^–6^	633	(59)
Ti_3_C_2_T_*x*_ and Ag NP complex	10^–8^	633	(60)
ordered Ag nanodot arrays deposited on ultrathin anodic aluminum oxide	5 × 10^–7^	785	(61)
Au NPs sputtered on G, GO, and rGO	10^–7^	514	(62)
Au NPs embedded in polymer matrix on G	<10^–10^	633	(63)
Ag NPs	10^–8^	532	(64)
Au NPs deposited on two-dimensional silicate nanoplatelets	10^–9^	532	(65)
Ag-coated capillary	10^–7^	633	(66)

a Abbreviations: G, graphene; GO, graphene oxide; and rGO, reduced graphene oxide.

The spectrum of bare Fe_3_O_4_@Ag is presented at the bottom of Figure 9A,B (0 M). Notably, it contains stretching modes of Fe–O and Ag–Cl, along with some extent of impurity modes. The disclosed adenine experiment shows the functionality of the nanoparticles and their ability to detect trace amounts of biologically relevant molecules outside of their electronic resonance. Additionally, the nanoparticles demonstrated long-term stability and effectiveness seven months after fabrication, assuming that they were adsorbed with chloride ions from the ablation solution.

## 4 Conclusions

Hybrid magneto-plasmonic nanoparticles (MPNPs) were synthesized by merging wet-chemically prepared Fe_3_O_4_ colloids with laser-ablated plasmonic nanoparticles (Au, Ag, or alloys). The magnetic and plasmonic properties acted in synergy to facilitate nanoparticle extraction, concentration, and targeted deposition while also enhancing molecular sensitivity via surface-enhanced Raman scattering (SERS). We quantified a SERS enhancement factor of 2 × 10^8^ at 633 nm laser excitation for Fe_3_O_4_@Ag using a 4-MBA molecular probe. Switching to Ag_*x*_Au_100–*x*_ alloys (*x* = 50 and 80 wt %) in the MPNP composition permitted the tuning of the plasmon resonance frequency but also led to a 10-fold SERS sensitivity drop compared to Fe_3_O_4_@Ag.

Furthermore, we correlated the chloride ion concentration in the ablation medium to the SERS sensitivity. Lower chloride concentrations (0.1–1 mM) amplified the SERS intensity across all nanoparticle compositions, while higher concentrations (20 mM) diminished the intensity for most of the nanoparticles. The MPNPs demonstrated long-term stability and functionality after a storage period of seven months at room temperature. Finally, the biologically relevant molecule adenine was probed to evaluate the Fe_3_O_4_@Ag nanoparticle SERS sensitivity. We determined the detection limit was 10^–7^ M and that there was a linear relationship between the spectral intensity and the logarithm of the adenine concentration in the 10^–3^ to 10^–7^ range. These findings have implications for developing MPNP-based SERS sensors for detecting trace amounts of biologically relevant molecules.

## Supporting Information Available

UV–vis spectra of Fe_3_O_4_@Ag and Fe_3_O_4_@Au NPs obtained by PLAL in pure and saline (30 mM) water, TEM EDX profiles of Fe_3_O_4_@Ag_80_Au_20_ and Fe_3_O_4_@Ag_50_Au_50_ NPs, and XRD patterns of Fe_3_O_4_@Ag_80_Au_20_ and Fe_3_O_4_@Ag_50_Au_50_ NPs (PDF)

## References

[ref1] Xu Z.; Hou Y.; Sun S. Magnetic core/shell Fe3O4/Au and Fe3O4/Au/Ag nanoparticles with tunable plasmonic properties. J. Am. Chem. Soc. 2007, 129, 8698–8699. 10.1021/ja073057v. 17590000

[ref2] Peng S.; Lei C.; Ren Y.; Cook R. E.; Sun Y. Plasmonic/magnetic bifunctional nanoparticles. Angew. Chemie - Int. Ed. 2011, 50, 3158–3163. 10.1002/anie.201007794. 21374772

[ref3] Levin C. S.; Hofmann C.; Ali T. A.; Kelly A. T.; Morosan E.; Nordlander P.; Whitmire K. H.; Halas N. J. Magnetic-Plasmonic Core-Shell Nanoparticles. ACS Nano 2009, 3, 1379–1388. 10.1021/nn900118a. 19441794 PMC4108303

[ref4] Kwizera E. A.; Chaffin E.; Shen X.; Chen J.; Zou Q.; Wu Z.; Gai Z.; Bhana S.; O'Connor R.; Wang L.; Adhikari H.; Mishra S. R.; Wang Y.; Huang X. Size- and Shape-Controlled Synthesis and Properties of Magnetic-Plasmonic Core-Shell Nanoparticles. J. Phys. Chem. C 2016, 120, 10530–10546. 10.1021/acs.jpcc.6b00875. PMC4882128 27239246

[ref5] Sirgedaite G.; Talaikis M.; Niaura G.; Mikoliunaite L. Magneto-plasmonic nanostructures for SERS: magnetite decorated by silver and gold nanoparticles. New J. Chem. 2022, 47, 402–411. 10.1039/D2NJ04368H.

[ref6] Eyvazzadeh N.; Shakeri-Zadeh A.; Fekrazad R.; Amini E.; Ghaznavi H.; Kamran Kamrava S. Gold-coated magnetic nanoparticle as a nanotheranostic agent for magnetic resonance imaging and photothermal therapy of cancer. Lasers Med. Sci. 2017, 32, 1469–1477. 10.1007/s10103-017-2267-x. 28674789

[ref7] Multari C.; Miola M.; Laviano F.; Gerbaldo R.; Pezzotti G.; Debellis D.; Verné E. Magnetoplasmonic nanoparticles for photothermal therapy. Nanotechnology. 2019, 30, 255705 10.1088/1361-6528/ab08f7. 30790778

[ref8] Michałowska A.; Kudelski A. The First Silver-Based Plasmonic Nanomaterial for Shell-Isolated Nanoparticle-Enhanced Raman Spectroscopy with Magnetic Properties. Molecules. 2022, 27, 3081 10.3390/molecules27103081. 35630560 PMC9143147

[ref9] Mikoliunaite L.; Talaikis M.; Michalowska A.; Dobilas J.; Stankevic V.; Kudelski A.; Niaura G. Thermally Stable Magneto-Plasmonic Nanoparticles for SERS with Tunable Plasmon Resonance. Nanomaterials 2022, 12, 2860 10.3390/nano12162860. 36014725 PMC9416134

[ref10] Michałowska A.; Krajczewski J.; Kudelski A. Magnetic iron oxide cores with attached gold nanostructures coated with a layer of silica: An easily, homogeneously deposited new nanomaterial for surface-enhanced Raman scattering measurements, Spectrochim. Acta - Part A Mol. Biomol. Spectrosc. 2022, 277, 121266 10.1016/j.saa.2022.121266. 35452900

[ref11] Berganza L. B.; Litti L.; Meneghetti M.; Lanceros-Méndez S.; Reguera J. Enhancement of Magnetic Surface-Enhanced Raman Scattering Detection by Tailoring Fe3O4@Au Nanorod Shell Thickness and Its Application in the On-site Detection of Antibiotics in Water. ACS Omega. 2022, 7, 45493–45503. 10.1021/acsomega.2c06099. 36530269 PMC9753213

[ref12] Petrikaitė V.; Skapas M.; Stankevičius E. Generation of gold and silver nanoparticles using laser ablation of thin bimetallic films and bulk targets in water. Opt. Mater. 2023, 137, 113535 10.1016/j.optmat.2023.113535.

[ref13] Torrisi L.; Torrisi A. Laser ablation parameters influencing gold nanoparticle synthesis in water. Radiat. Eff. Defects Solids. 2018, 173, 729–739. 10.1080/10420150.2018.1528598.

[ref14] Petrikaitė V.; Ignatjev I.; Selskis A.; Niaura G.; Stankevičius E. Hybrid gold-silver nanoparticles synthesis on a glass substrate using a nanosecond laser-induced dewetting of thin bimetallic films and their application in SERS. Opt. Laser Technol. 2024, 168, 109956 10.1016/j.optlastec.2023.109956.

[ref15] Stankevičius E.; Garliauskas M.; Laurinavičius L.; Trusovas R.; Tarasenko N.; Pauliukaitė R. Engineering electrochemical sensors using nanosecond laser treatment of thin gold film on ITO glass. Electrochim. Acta 2019, 297, 511–522. 10.1016/j.electacta.2018.11.197.

[ref16] Tarasenka N.; Kiris V.; Stankevičius E.; Tarasenko N.; Pankov V.; Krčma F.; Gečys P.; Račiukaitis G. Laser Irradiation of Gd–Si and Gd–Si–Ge Colloid Mixtures for the Fabrication of Compound Nanoparticles. ChemPhysChem. 2018, 19, 3247–3256. 10.1002/cphc.201800753. 30285314

[ref17] Amendola V.; Meneghetti M. Laser ablation synthesis in solution and size manipulation of noble metal nanoparticles. Phys. Chem. Chem. Phys. 2009, 11, 3805–3821. 10.1039/b900654k. 19440607

[ref18] Shih C. Y.; Shugaev M. V.; Wu C.; Zhigilei L. V. The effect of pulse duration on nanoparticle generation in pulsed laser ablation in liquids: Insights from large-scale atomistic simulations. Phys. Chem. Chem. Phys. 2020, 22, 7077–7099. 10.1039/D0CP00608D. 32196057

[ref19] Dell’Aglio M.; Gaudiuso R.; De Pascale O.; De Giacomo A. Mechanisms and processes of pulsed laser ablation in liquids during nanoparticle production. Appl. Surf. Sci. 2015, 348, 4–9. 10.1016/j.apsusc.2015.01.082.

[ref20] Subhan A.; Mourad A. H. I.; Al-Douri Y. Influence of Laser Process Parameters, Liquid Medium, and External Field on the Synthesis of Colloidal Metal Nanoparticles Using Pulsed Laser Ablation in Liquid: A Review. Nanomaterials 2022, 12 (13), 2144 10.3390/nano12132144. 35807980 PMC9268572

[ref21] Naser H.; Alghoul M.A.; Hossain M.K.; Asim N.; Abdullah M.F.; Ali M.S.; Alzubi F.G.; Amin N. The role of laser ablation technique parameters in synthesis of nanoparticles from different target types. J. Nanoparticle Res. 2019, 21, 249 10.1007/s11051-019-4690-3.

[ref22] Lévy A.; De Anda Villa M.; Laurens G.; Blanchet V.; Bozek J.; Gaudin J.; Lamour E.; Macé S.; Mignon P.; Milosavljević A. R.; Nicolas C.; Patanen M.; Prigent C.; Robert E.; Steydli S.; Trassinelli M.; Vernhet D.; Veteläinen O.; Amans D. Surface Chemistry of Gold Nanoparticles Produced by Laser Ablation in Pure and Saline Water. Langmuir. 2021, 37, 5783–5794. 10.1021/acs.langmuir.1c00092. 33939435

[ref23] Zhou Z. M.; Zheng H.; Liu T.; Xie Z. Z.; Luo S. H.; Chen G. Y.; Tian Z. Q.; Liu G. K. Improving SERS Sensitivity toward Trace Sulfonamides: The Key Role of Trade-Off Interfacial Interactions among the Target Molecules, Anions, and Cations on the SERS Active Surface. Anal. Chem. 2021, 93, 8603–8612. 10.1021/acs.analchem.1c01530. 34115465

[ref24] Dong X.; Gu H.; Liu F. Effect of halide ions on the surface-enhanced Raman spectroscopy of methylene blue for borohydride-reduced silver colloid. J. Phys. Conf. Ser. 2011, 277, 012030 10.1088/1742-6596/277/1/012030.

[ref25] Xie L.; Lu J.; Liu T.; Chen G.; Liu G.; Ren B.; Tian Z. Key Role of Direct Adsorption on SERS Sensitivity: Synergistic Effect among Target, Aggregating Agent, and Surface with Au or Ag Colloid as Surface-Enhanced Raman Spectroscopy Substrate. J. Phys. Chem. Lett. 2020, 11, 1022–1029. 10.1021/acs.jpclett.9b03724. 31931563

[ref26] Koo T.-W.; Chan S.; Sun L.; Su X.; Zhang J.; Berlin A. A. Specific chemical effects on surface-enhanced Raman spectroscopy for ultra-sensitive detection of biological molecules. Appl. Spectrosc. 2004, 58, 1401–1407. 10.1366/0003702042641227. 15606951

[ref27] Pang R.; Zhang X. G.; Zhou J. Z.; Wu D. Y.; Tian Z. Q. SERS Chemical Enhancement of Water Molecules from Halide Ion Coadsorption and Photoinduced Charge Transfer on Silver Electrodes. J. Phys. Chem. C 2017, 121, 10445–10454. 10.1021/acs.jpcc.7b02408.

[ref28] Muniz-Miranda M.; Muniz-Miranda F.; Giorgetti E. Spectroscopic and microscopic analyses of Fe3O4/au nanoparticles obtained by laser ablation in water. Nanomaterials 2020, 10 (1), 132 10.3390/nano10010132. 31936852 PMC7023500

[ref29] Tymoczko A.; Kamp M.; Rehbock C.; Kienle L.; Cattaruzza E.; Barcikowski S.; Amendola V. One-step synthesis of Fe-Au core-shell magnetic-plasmonic nanoparticles driven by interface energy minimization. Nanoscale Horizons. 2019, 4, 1326–1332. 10.1039/C9NH00332K.

[ref30] Kamp M.; Tymoczko A.; Popescu R.; Schürmann U.; Nadarajah R.; Gökce B.; Rehbock C.; Gerthsen D.; Barcikowski S.; Kienle L. Composition and structure of magnetic high-temperature-phase, stable Fe-Au core-shell nanoparticles with zero-valent bcc Fe core. Nanoscale Adv. 2020, 2, 3912–3920. 10.1039/D0NA00514B. 36132793 PMC9417649

[ref31] Mikoliunaite L.; Stankevičius E.; Adomavičiu̅tė-Grabusovė S.; Petrikaitė V.; Trusovas R.; Talaikis M.; Skapas M.; Zdaniauskienė A.; Selskis A.; Šablinskas V.; Niaura G. Magneto-Plasmonic Nanoparticles Generated by Laser Ablation of Layered Fe/Au and Fe/Au/Fe Composite Films for SERS Application. Coatings 2023, 13, 1523 10.3390/coatings13091523.

[ref32] Amendola V.; Scaramuzza S.; Carraro F.; Cattaruzza E. Formation of alloy nanoparticles by laser ablation of Au/Fe multilayer films in liquid environment. J. Colloid Interface Sci. 2017, 489, 18–27. 10.1016/j.jcis.2016.10.023. 27770998

[ref33] Amendola V.; Scaramuzza S.; Litti L.; Meneghetti M.; Zuccolotto G.; Rosato A.; Nicolato E.; Marzola P.; Fracasso G.; Anselmi C.; Pinto M.; Colombatti M. Magneto-plasmonic Au-Fe alloy nanoparticles designed for multimodal SERS-MRI-CT imaging. Small. 2014, 10, 2476–2486. 10.1002/smll.201303372. 24619736

[ref34] Amendola V.; Meneghetti M.; Bakr O. M.; Riello P.; Polizzi S.; Anjum D. H.; Fiameni S.; Arosio P.; Orlando T.; De Julian Fernandez C.; Pineider F.; Sangregorio C.; Lascialfari A. Coexistence of plasmonic and magnetic properties in Au89Fe 11 nanoalloys. Nanoscale. 2013, 5, 5611–5619. 10.1039/c3nr01119d. 23685617

[ref35] Michałowska A.; Żygieło M.; Kudelski A. Fe3O4-protected gold nanoparticles: New plasmonic-magnetic nanomaterial for Raman analysis of surfaces. Appl. Surf. Sci. 2021, 562, 150220 10.1016/j.apsusc.2021.150220.

[ref36] Aleknavičienė I.; Pabrėža E.; Talaikis M.; Jankunec M.; Račiukaitis G. Low-cost SERS substrate featuring laser-ablated amorphous nanostructure. Appl. Surf. Sci. 2022, 571, 151248 10.1016/j.apsusc.2021.151248.

[ref37] Khinevich N.; Juodėnas M.; Tamulevičienė A.; Tamulevičius T.; Talaikis M.; Niaura G.; Tamulevičius S. Wavelength-tailored enhancement of Raman scattering on a resonant plasmonic lattice. Sensors Actuators B Chem. 2023, 394, 134418 10.1016/j.snb.2023.134418.

[ref38] Sisco P. N.; Murphy C. J. Surface-coverage dependence of surface-enhanced Raman scattering from gold nanocubes on self-asssembled monolayers of analyte. J. Phys. Chem. A 2009, 113, 3973–3978. 10.1021/jp810329j. 19271748

[ref39] Zhang Q.; Lee J.Y.; Yang J.; Boothroyd C.; Zhang J. Size and composition tunable Ag-Au alloy nanoparticles by replacement reactions. Nanotechnology 2007, 18, 245605 10.1088/0957-4484/18/24/245605.

[ref40] Sun L.; Luan W.; Shan Y. J. A composition and size controllable approach for Au-Ag alloy nanoparticles. Nanoscale Res. Lett. 2012, 7, 1–6. 10.1186/1556-276X-7-225. 22513005 PMC3487982

[ref41] Bell S. E. J.; Charron G.; Cortés E.; Kneipp J.; de la Chapelle M. L.; Langer J.; Procházka M.; Tran V.; Schlücker S. Towards Reliable and Quantitative Surface-Enhanced Raman Scattering (SERS): From Key Parameters to Good Analytical Practice. Angew. Chemie - Int. Ed. 2020, 59, 5454–5462. 10.1002/anie.201908154. PMC7154527 31588641

[ref42] Zambzickaite G.; Talaikis M.; Dobilas J.; Stankevic V.; Drabavicius A.; Niaura G.; Mikoliunaite L. Microwave-Assisted Solvothermal Synthesis of Nanocrystallite-Derived Magnetite Spheres. Materials 2022, 15, 4008 10.3390/ma15114008. 35683306 PMC9181964

[ref43] Daublytė E.; Zdaniauskienė A.; Talaikis M.; Drabavičius A.; Charkova T. A facile microwave-assisted synthesis of Ag@SiO 2 nanoparticles for Raman spectroscopy. New J. Chem. 2021, 45, 10952–10958. 10.1039/D1NJ01439K.

[ref44] Michota A.; Bukowska J. Surface-enhanced Raman scattering (SERS) of 4-mercaptobenzoic acid on silver and gold substrates. J. Raman Spectrosc. 2003, 34, 21–25. 10.1002/jrs.928.

[ref45] Capocefalo A.; Mammucari D.; Brasili F.; Fasolato C.; Bordi F.; Postorino P.; Domenici F. Exploring the potentiality of a SERS-active pH nano-biosensor. Front. Chem. 2019, 7, 1–11. 10.3389/fchem.2019.00413. 31231638 PMC6568054

[ref46] Golubewa L.; Karpicz R.; Matulaitiene I.; Selskis A.; Rutkauskas D.; Pushkarchuk A.; Khlopina T.; Michels D.; Lyakhov D.; Kulahava T.; Shah A.; Svirko Y.; Kuzhir P. Surface-Enhanced Raman Spectroscopy of Organic Molecules and Living Cells with Gold-Plated Black Silicon. ACS Appl. Mater. Interfaces. 2020, 12, 50971–50984. 10.1021/acsami.0c13570. 33107725

[ref47] Kundu J.; Neumann O.; Janesko B. G.; Zhang D.; Lal S.; Barhoumi A.; Scuseria G. E.; Halas N. J. Adenine-and adenosine monophosphate (AMP)-gold binding interactions studied by surface-enhanced Raman and infrared spectroscopies. J. Phys. Chem. C 2009, 113, 14390–14397. 10.1021/jp903126f.

[ref48] Huang R.; Yang H. T.; Cui L.; Wu D. Y.; Ren B.; Tian Z. Q. Structural and charge sensitivity of surface-enhanced Raman spectroscopy of adenine on silver surface: A quantum chemical study. J. Phys. Chem. C 2013, 117, 23730–23737. 10.1021/jp407615r.

[ref49] Yao G.; Zhai Z.; Zhong J.; Huang Q. DFT and SERS Study of 15N Full-Labeled Adenine Adsorption on Silver and Gold Surfaces. J. Phys. Chem. C 2017, 121, 9869–9878. 10.1021/acs.jpcc.7b00818.

[ref50] Testa-Anta M.; Ramos-Docampo M. A.; Comesaña-Hermo M.; Rivas-Murias B.; Salgueiriño V. Raman spectroscopy to unravel the magnetic properties of iron oxide nanocrystals for bio-related applications. Nanoscale Adv. 2019, 1, 2086–2103. 10.1039/C9NA00064J. 36131987 PMC9418671

[ref51] Alula M. T.; Yang J. Photochemical decoration of silver nanoparticles on magnetic microspheres as substrates for the detection of adenine by surface-enhanced Raman scattering. Anal. Chim. Acta 2014, 812, 114–120. 10.1016/j.aca.2013.12.028. 24491771

[ref52] Tzeng Y.; Lin B.-Y. Silver SERS adenine sensors with a very low detection limit. Biosensors 2020, 10 (5), 53 10.3390/bios10050053. 32429203 PMC7277772

[ref53] Gao D.; Yang X.; Teng P.; Kong D.; Liu Z.; Yang J.; Luo M.; Li Z.; Wen X.; Yuan L.; Li K.; Bowkett M.; Copner N.; Wang X. On-line SERS detection of adenine in DNA based on the optofluidic in-fiber integrated GO/PDDA/Ag NPs. Sensors Actuators, B Chem. 2021, 332, 129517 10.1016/j.snb.2021.129517.

[ref54] Wu P. F.; Fan X. Y.; Xi H. Y.; Pan N.; Shi Z. Q.; You T. T.; Gao Y. K.; Yin P. G. Multifunctional self-assembled gold nanorod monolayer/Ti3C2Tx nanocomposites based on interfacial electrostatic for highly sensitive SERS detection of organic dyes and adenine. J. Alloys Compd. 2022, 920, 165978 10.1016/j.jallcom.2022.165978.

[ref55] Tegegne W. A.; Su W. N.; Beyene A. B.; Huang W. H.; Tsai M. C.; Hwang B. J. Flexible hydrophobic filter paper-based SERS substrate using silver nanocubes for sensitive and rapid detection of adenine. Microchem. J. 2021, 168, 106349 10.1016/j.microc.2021.106349.

[ref56] Zhang K.; Yao S.; Li G.; Hu Y. One-step sonoelectrochemical fabrication of gold nanoparticle/carbon nanosheet hybrids for efficient surface-enhanced Raman scattering. Nanoscale. 2015, 7, 2659–2666. 10.1039/C4NR07082H. 25580806

[ref57] Lai H.; Zhang H.; Li G.; Hu Y. Bimetallic AgNPs@dopamine modified-halloysite nanotubes-AuNPs for adenine determination using surface-enhanced Raman scattering. Microchim. Acta. 2021, 188, 1–11. 10.1007/s00604-021-04778-1. 33733686

[ref58] Zhu Y.; Dluhy R. A.; Zhao Y. Development of silver nanorod array based fiber optic probes for SERS detection. Sensors Actuators, B Chem. 2011, 157, 42–50. 10.1016/j.snb.2011.03.024.

[ref59] Lee J. W.; Kim K.; Shin K. S. A novel fabrication of Au-coated glass capillaries for chemical analysis by surface-enhanced Raman scattering. Vib. Spectrosc. 2010, 53, 121–125. 10.1016/j.vibspec.2010.01.002.

[ref60] Liu R.; Jiang L.; Yu Z.; Jing X.; Liang X.; Wang D.; Yang B.; Lu C.; Zhou W.; Jin S. MXene (Ti3C2Tx)-Ag nanocomplex as efficient and quantitative SERS biosensor platform by in-situ PDDA electrostatic self-assembly synthesis strategy. Sensors Actuators, B Chem. 2021, 333, 129581 10.1016/j.snb.2021.129581.

[ref61] Chen G.; Wang Y.; Wang H.; Cong M.; Chen L.; Yang Y.; Geng Y.; Li H.; Xu S.; Xu W. A highly sensitive microfluidics system for multiplexed surface-enhanced Raman scattering (SERS) detection based on Ag nanodot arrays. RSC Adv. 2014, 4, 54434–54440. 10.1039/C4RA09251A.

[ref62] Liang X.; Liang B.; Pan Z.; Lang X.; Zhang Y.; Wang G.; Yin P.; Guo L. Tuning plasmonic and chemical enhancement for SERS detection on graphene-based Au hybrids. Nanoscale. 2015, 7, 20188–20196. 10.1039/C5NR06010A. 26575834

[ref63] Juang R. S.; Wang K. S.; Cheng Y. W.; Fu C. C.; Chen W. T.; Liu C. M.; Chien C. C.; Jeng R. J.; Chen C. C.; Liu T. Y. Floating SERS substrates of silver nanoparticles-graphene based nanosheets for rapid detection of biomolecules and clinical uremic toxins. Colloids Surfaces A Physicochem. Eng. Asp. 2019, 576, 36–42. 10.1016/j.colsurfa.2019.05.042.

[ref64] Sivaprakasam V.; Hart M. B. Surface-Enhanced Raman Spectroscopy for Environmental Monitoring of Aerosols. ACS Omega. 2021, 6, 10150–10159. 10.1021/acsomega.1c00207. 34056169 PMC8153665

[ref65] Lee Y. C.; Chiu C.-W. Immobilization and 3D hot-junction formation of gold nanoparticles on two-dimensional silicate nanoplatelets as substrates for high-efficiency surface-enhanced Raman scattering detection. Nanomaterials 2019, 9 (3), 324 10.3390/nano9030324. 30823691 PMC6473534

[ref66] Park H. K.; Lee H. B.; Kim K. A facile deposition of silver onto the inner surface of a glass capillary tube for micro-surface-enhanced Raman scattering measurements. Appl. Spectrosc. 2007, 61, 19–24. 10.1366/000370207779701325. 17311712

