# Supplementary material for: Multiwavelength SERS of Magneto-Plasmonic Nanoparticles Obtained by Combined Laser Ablation and Solvothermal Methods
Source: ACS Omega. 2023 Dec 14;8(51):49396–405. doi: 10.1021/acsomega.3c08007 (PMC10753541; doi:10.1021/acsomega.3c08007)
Supplement: Supplementary file 1 — ao3c08007_si_001.pdf [file ao3c08007_si_001.pdf]

SUPPLEMENTARY INFORMATION

for

Multiwavelength SERS of Magneto-Plasmonic Nanoparticles Obtained by Combined Laser Ablation and Solvothermal Methods

Martynas Talaikis[1*], Lina Mikoliunaite[1, 2], Aikaterini-Maria Gkouzi[1], Vita Petrikaitė[3], Evaldas Stankevičius[3], Audrius Drabavičius[4], Algirdas Selskis[4], Remigijus Juškėnas[4], Gediminas Niaura[1]

[1] Department of Organic Chemistry, Center for Physical Sciences and Technology (FTMC), Sauletekio Av. 3, LT-10257 Vilnius, Lithuania;

[2] Department of Physical Chemistry, Faculty of Chemistry and Geosciences, Vilnius University, Naugarduko Str. 24, LT-03225 Vilnius, Lithuania;

[3] Department of Laser Technologies, Center for Physical Sciences and Technology (FTMC), Savanoriu Av. 231, LT-02300 Vilnius, Lithuania;

[4] Department of Characterization of Materials Structure, Center for Physical Sciences and Technology (FTMC), Sauletekio Av. 3, LT-10257 Vilnius, Lithuania;

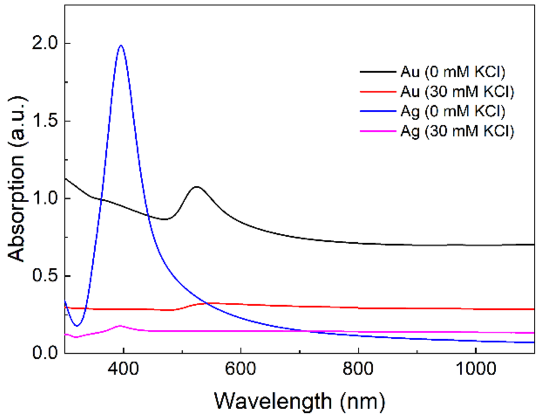

Figure S1. Comparison of UV-Vis spectra of Fe_3O_4@Ag and Fe_3O_4@Au NPs obtained by PLAL in pure and saline (30 mM) water.

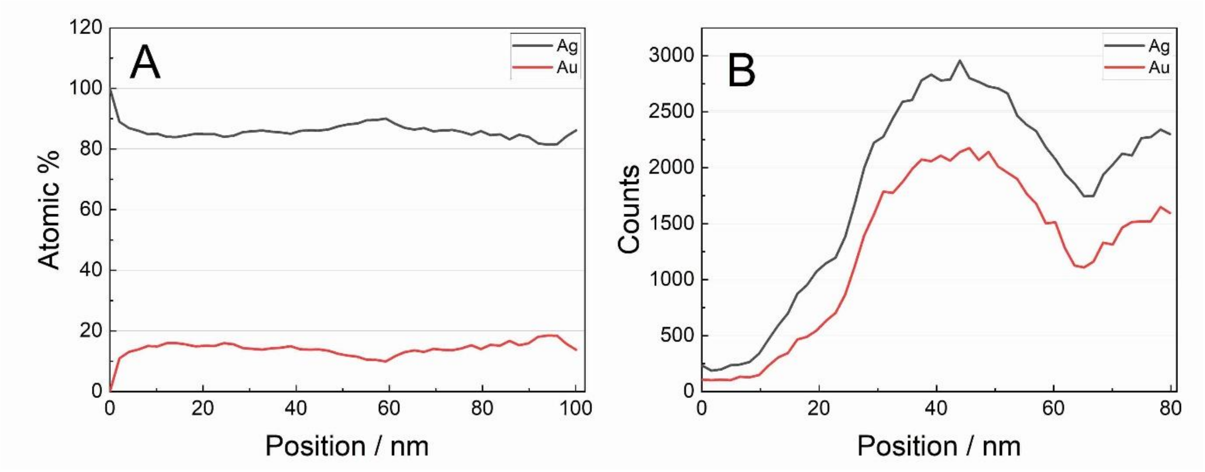

Figure S2. (A) TEM EDX profiles of Fe_3O_4@$Ag_{80}Au_{20}$ and (B) Fe_3O_4@$Ag_{50}Au_{50}$ NPs.

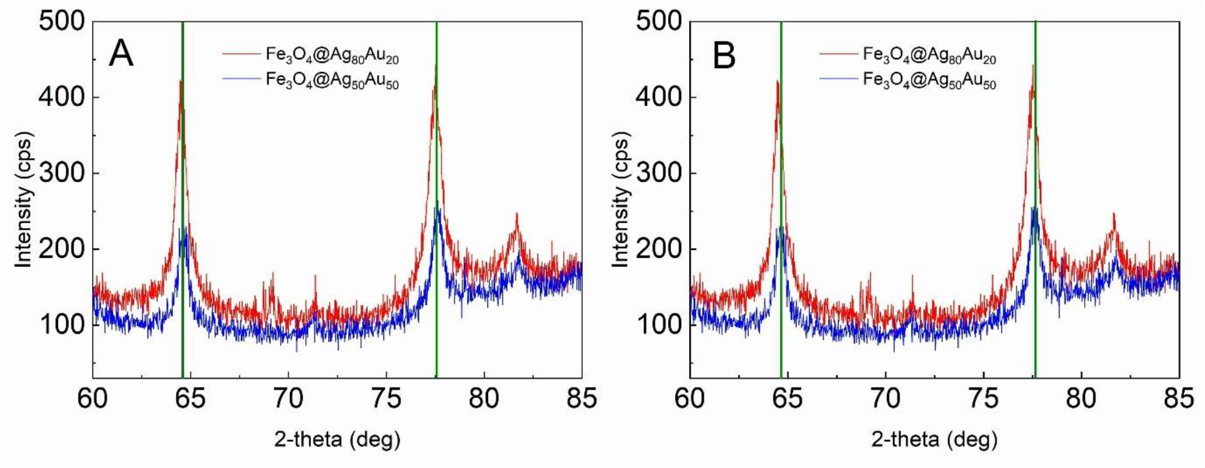

Figure S3. XRD patterns of Fe_3O_4@$Ag_{80}Au_{20}$ (red curve) and Fe_3O_4@$Ag_{50}Au_{50}$ (blue curve) NPs. The green line marks theoretical peaks positions of (A) $Ag_{75}Au_{25}$ and (B) $Ag_{50}Au_{50}$ standards. The theoretical lines quite well match experimentally obtained spectral peaks.
